# AG490 protects cerebral ischemia/reperfusion injury via inhibiting the JAK2/3 signaling pathway

**DOI:** 10.1002/brb3.1911

**Published:** 2020-10-23

**Authors:** Lichao Fan, Lichun Zhou

**Affiliations:** ^1^ Department of Neurology Beijing Chaoyang Hospital Capital Medical University Beijing China

**Keywords:** AG490, apoptosis, Cerebral ischemia/reperfusion injury, JAK2, MCAO

## Abstract

**Background:**

Cerebral ischemia/reperfusion injury is a severe problem in patients with brain ischemia. Brain injury caused by the immune response is important in the pathogenesis of cerebral ischemia/reperfusion injury and immune pathways. It is important to investigate potential targets for the treatment of cerebral ischemia/reperfusion injury.

**Methods:**

In this experiment, we evaluated the effect of an exogenous JAK antagonist AG490 in the cerebral ischemia/reperfusion injury model, which was established by middle cerebral artery occlusion (MCAO). Histology study, TUNEL staining, Western blot, and RT‐PCR were employed to examine the effects of AG490 in cerebral ischemia/reperfusion injury.

**Results:**

In the brain tissue of MCAO mice, JAK2 was highly expressed. AG490 is an inhibitor of JAK2, which reduced the phosphorylation level of JAK2. AG490 downregulated the phosphorylated activation of JAK3 and their downstream STAT3. The antiapoptotic activity of AG490 on cerebral ischemia/reperfusion injury mice was consistent with in vitro data. It reduced the phosphorylation of JAK2/JAK3/STAT3 and the apoptosis rate in cultured neurons upon apoptosis induction. Besides, we also observed the neuroprotective effects of AG490 on cerebral ischemia/reperfusion injury. Administration of AG490 could further enhance the expression of neurotrophins including BNDF, NT3, and the neurotrophin receptor TrkB.

**Conclusion:**

Therefore, AG490 is pluripotent for cerebral ischemia/reperfusion injury through both antiapoptosis and neuroprotective activities. The antiapoptosis effect is dependent on its regulation of the JAK‐STAT pathway.

## Graphical abstract

AG490 injection could attenuate the lesions indicated by histology study. AG490 exhibited antiapoptotic activity indicated by TUNEL staining. AG490 antiapoptotic activity on CIRI rats was consistent with in vitro data, reducing the phosphorylation of JAK2/JAK3/STAT3 and the apoptotic rate in cultured neuron upon apoptosis induction.

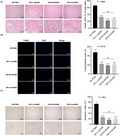

## 1 INTRODUCTION

It is well established that the antiapoptotic effects have essential protective effects on cerebral ischemia models in vivo (Zhang et al., (2019); Briyal et al., 2019). Extracellular stimuli and specific receptor interaction at the cell surface can lead to the activation of kinases of the Janus kinase (JAK) family and phosphorylate substrate proteins of signal transducers and activators of transcription (STATs). AG490 was reported to downregulate the mitogen‐activated protein kinase (MAPK) and JAK‐STAT pathways for apoptosis inducement in myeloma cells, which might be beneficial to antitumor activity (De Vos et al., 2000; Sun et al., 2001). However, the roles of AG490 on neural cells apoptosis are unknown. In this study, we will investigate the effect of AG490 on the mouse MCAO model of cerebral ischemia/reperfusion injury.

The phosphorylated STAT proteins could move to the nucleus, bind specific DNA elements, and have direct transcription (Frank & Varticovski, 1996; Mertens & Darnell, 2007). JAK‐STAT signaling dysfunction is associated with various human diseases ( Rawlings et al., 2004; Schindler et al., 2007). Accumulative evidence sheds light on the precise mechanism of JAK‐STAT pathway triggered by a variety of cytokines (Schindler et al., (2007); Heinrich et al., 1998). Different apoptosis or anti‐apoptosis‐related genes can also be transcriptionally modulated via JAK‐STAT upon upstream receptor activation (Jiang, et al., 2009a; Liu et al., 1998). For instance, a member of the TNF receptor superfamily can transduce signal through JAK‐STAT, which may induce tumor cell apoptosis (Jiang, et al., 2009a; Liu et al., 1998).

The selectively targeting JAK‐STAT by exogenously synthesized or intrinsic small molecule inhibitors could result in the apoptosis alternation both in normal cells and in tumor cells (O'Sullivan et al., 2007; Schindler, 1999). It was reported that activities of JAK1 and JAK2, the upstream kinases, are essential for STAT1 signaling in response to IFN‐gamma (Chen et al., (2002); Rui et al., 1998). It could be reduced by an endonuclease inhibitor aurintricarboxylic acid (Chen et al., 2002; Rui et al., 1998). miR‐340 affected gastric cancer cell apoptosis by regulating the SOCS3/JAK‐STAT signaling pathway (Xiao et al., 2018). JAB/SOCS‐1 has been shown to be an intrinsic JAK tyrosine kinase inhibitor, which could suppress the cytokine‐dependent JAK‐STAT pathway (Iwamoto et al., 2000). The pro‐apoptotic and antiapoptotic efficacy of molecular intervention in JAK‐STAT pathway depends on different inhibitors and cell types (Jiang et al., 2009b; Negoro et al., 2000). In the present study, we will evaluate the role of a selective JAK inhibitor AG490 on middle cerebral artery occlusion (MCAO)‐induced cerebral ischemia/reperfusion injury in mice.

## 2 MATERIALS AND METHODS

### 2.1 Mouse MCAO Model

C57 mice (6 weeks old, 18–22 g) were subjected to 2 hr of MCAO followed by 24 hr of reperfusion. The mice were anesthetized with sodium pentobarbital (50 mg/kg, i.p.). The diameter of thread was about 0.235 mm. The thread was inserted from the common carotid artery to the right internal carotid artery (ICA) until it passed the middle cerebral artery (MCA) origin with approximately 20 mm. After reperfusion for 24 hr, the mice were sacrificed, and the brains were harvested. The cerebral infarct volume was evaluated by using 2,3,5‐triphenyltetrazolium chloride (TTC) staining. Another specimen was homogenized in ice‐cold normal saline by 1:9 (w/v) for the assays of biochemical indexes. For AG490 treatment, different concentrations of AG490 were administered intraperitoneally (i.p.) after ischemia/reperfusion (I/R) operation. The contents of AG490 (APExBio, China, #A4139) were set up as low concentration (5 μM), middle concentration (10 μM), and high concentration (15 μM) according to the reported IC50 value for JAK2 (Wang et al., 1999). This study was approved by the animal experimental ethics committee of Beijing Chaoyang Hospital, Capital Medical University (reference number: AEEI‐2020‐090), and conducted in strict accordance with the National Institutes of Health Guidelines for the Care and Use of Experimental Animals.

### 2.2 Cell treatment

After the mice were anesthetized with sodium pentobarbital, the skin of the abdomen was disinfected with iodine and 75% ethanol. The cerebral cortex was isolated under a dissecting microscope. We cut the tissue, added trypsin‐EDTA digestion solution, and placed it in a 37°C incubator for 20 min. Then, it was centrifuged in prechilled culture medium (DMEM + 10% FBS) to stop the digestion for 5 min. The supernatant was collected and filtered through a 200‐mesh screen. The filtered cell suspension was centrifuged at 800 rpm for 5 min. Then, the supernatant was discarded. Cells were counted, and the density was adjusted to 1.5 × 10^5^/cm^2^. For experiment, lipopolysaccharide was used to induce cell apoptosis. AG490 (APExBio, China, #A4139) was added to treat the cells. The apoptosis and related protein expressions were determined by flow cytometry and Western blot, respectively.

### 2.3 RNA isolation and real‐time PCR

Total RNA was extracted from cells using TRIzol reagent (Invitrogen, San Diego, CA, USA) in accordance with the manufacturer's instructions. Reverse transcription and quantitative real‐time PCR using SYBR Green were performed to compare the relative expression levels of specific mRNAs. The cycling program involved preliminary denaturation at 95°C for 5 min, followed by 40 cycles of denaturation at 95°C for 15 s, annealing at 60°C for 30 s, and elongation at 72°C for 30 s, followed by a final elongation step at 72°C for 5 min. The relative mRNA levels of each gene were normalized to β‐actin as described previously. Real‐time PCR measurements were performed to obtain a mean CT value for each sample. The CT values of the different samples were compared using the 2^−ΔΔCT^ method. Actin expression levels were used as an internal reference.

### 2.4 Western blot

Briefly, equal amounts of proteins were separated from a polyacrylamide gel and electro‐transferred to polyvinylidene fluoride membranes (Millipore Corp.). Membranes were blocked overnight with the primary antibodies. After incubating with the secondary antibodies, the membranes were washed five times with TBST (pH 7.6) for 30 min at room temperature. Chemiluminescence reagent SuperSignal™ West Femto Substrate (Thermo Fisher Scientific) was used to develop the membranes according to the manufacturer's instruction. Immunoreactive bands were visualized using the BIO‐RAD ChemiDoc XRS Imaging System according to the manufacturer's instruction. Densitometric analysis was performed with Quantity One software (Bio‐Rad). The antibodies used were anti‐TNFa (Abcam, #ab66579), anti‐FAS (Abcam, #ab82419), anti‐Bax (Proteintech, #50599‐2‐Ig), anti‐Bcl‐2 (Abcam, #ab196495), anti‐cleaved caspase‐3 (Abcam, #ab49822), anti‐IL6 (Abcam, #ab208113), anti‐Akt (Cell Signaling Technology, #4685), anti‐p‐Akt (Cell Signaling Technology, #4060), anti‐beta‐actin (Proteintech, 60008‐1‐Ig), anti‐P‐BAD (Abcam, ab129192), anti‐p‐Bax (Abcam, ab111391), and anti‐p‐Par4 (Cell Signaling Technology, #2329).

### 2.5 Terminal deoxynucleotidyl‐transferase mediated dUTP‐biotin nick end‐labeling (TUNEL) staining

Cell apoptosis was determined in tissues fixed by TUNEL assay through an In Situ Cell Death Detection Kit (Roche, Germany). Immunofluorescent‐labeled TUNEL‐positive cells were recorded by a color digital camera (Olympus).

### 2.6 Flow cytometry

Cell apoptosis was measured through Annexin V‐FITC (Thermo Fisher Scientific, USA). Cells were harvested and washed in phosphate buffer saline (PBS) twice. Cells were then incubated and treated by Annexin V‐FITC and PI at 25 Celsius for 15 min in the dark. The apoptotic cells were analyzed by a FACScan flow cytometer (BD Biosciences, USA).

### 2.7 Hematoxylin–eosin (H&E) staining

Preoptic area (POA) tissues were fixed with 4% formaldehyde, embedded in paraffin wax, and cut into 3‐μm slices. The tissues were dehydrated, dewaxed, and then treated in gradient ethanol for 5 min. Then, they were rehydrated. We stained the slices with hematoxylin for 10 min. After washing, the slices were stained by eosin. Images were taken at 100× and 400× magnification.

### 2.8 Statistical analyses

All our results were expressed as the mean ± standard deviation (*SD*). Data were compared with independent‐samples *t* test between two groups and one‐way analysis of variance (ANOVA) among multiple groups, followed by Tukey's post hoc tests. There were 5 mice in each group. All experiments were repeated three times. *p* < .05 was considered to be significant.

## 3 RESULTS

### 3.1 *AG490 exerted protection effects by preventing the neural apoptosis* in vivo

**Figure 1:**
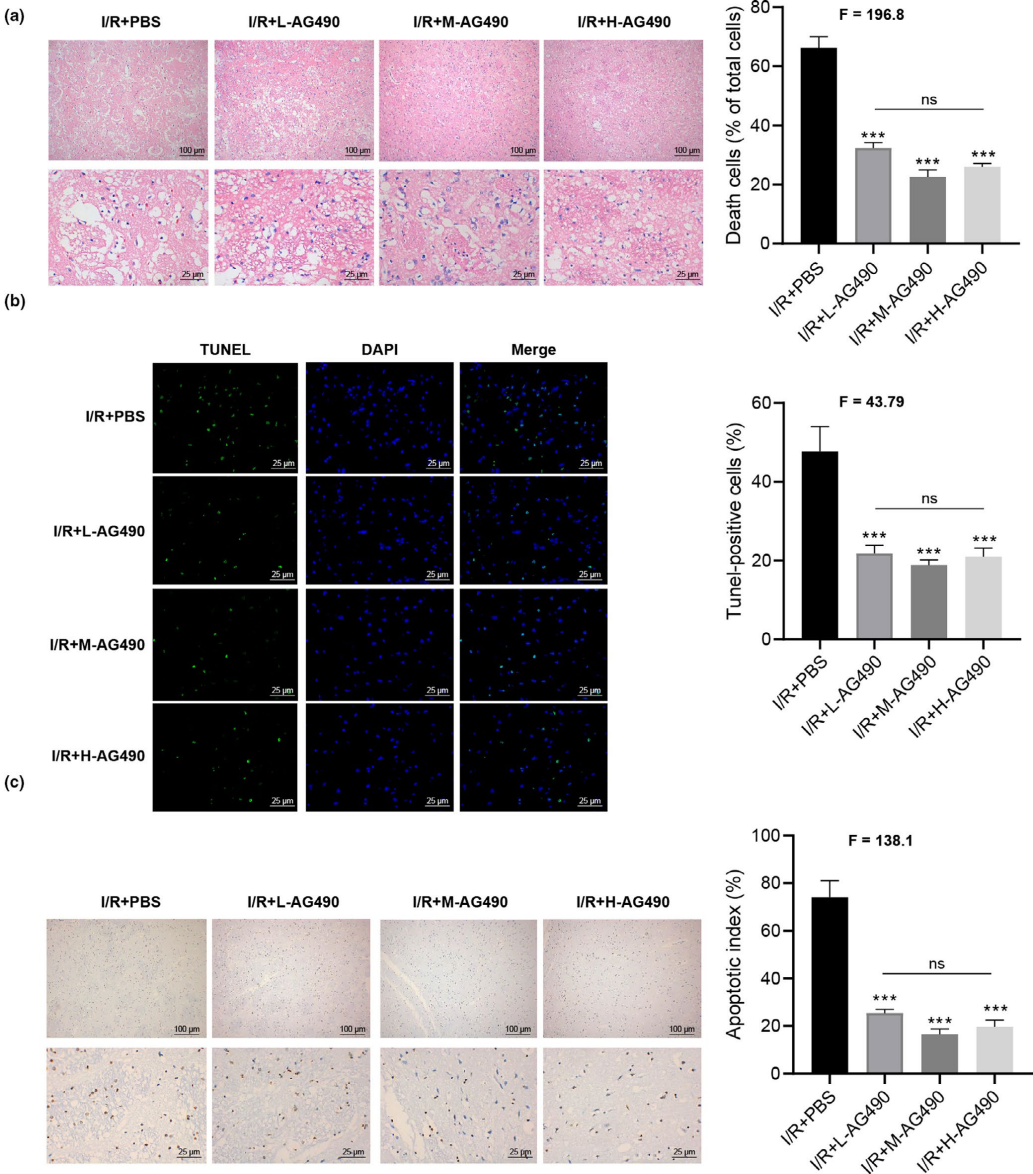
AG490 exerted protection effects by preventing the neural apoptosis in vivo. (a). H.E staining displays the change in preoptic area. I/R + PBS, I/R + L‐AG490, I/R + M‐AG490, and I/R + H‐AG490. (b). AG490 prevented the neural apoptosis demonstrated by TUNEL staining. (c). AG490 prevented significant cell apoptosis by TUNEL staining, in comparison with I/R + PBS (above panel: 100 × magnification; below panel: 400× magnification) from the I/R + PBS and I/R + L‐AG490, I/R + M‐AG490, and I/R + H‐AG490 groups. One‐way ANOVA was used among multiple groups. *n* = 5 for each group. ns, not significant; *p < .05, **p < .01, ***p < .001 compared with I/R + PBS

The cerebral ischemia/reperfusion injury was established by the MCAO method. JAK/STAT inhibitor AG490 was injected intraperitoneally. The POA was observed. Severe cerebral infarction induced by MCAO was observed, while the I/R + AG490 group showed no neurological defects. In morphology, the MCAO group with AG490 injection at different concentrations could ameliorate the inflammatory reaction in the infarction sites indicated by H.E staining, and the cell death rate was lower (*p* < .05), compared with the vehicle injection group (Figure 1a). In particular, low, medium, and high concentrations of AG490 also prevented the neural apoptosis demonstrated by TUNEL staining from the data point of view (Figure 1b). Therefore, AG490 is beneficial for the MCAO‐induced cerebral ischemia/reperfusion injury in vivo. From Figure 1c, it was obvious that I/R + PBS caused significant cell apoptosis by TUNEL staining (*p* < .05). The above results indicated that there is no a dosage‐dependent effect from AG490 in vivo. It also supported that AG490 exerted protection effects by preventing the neural apoptosis in vivo.

### 3.2 AG490 regulated the phosphorylation of JAK2/3 and STAT3

**Figure 2:**
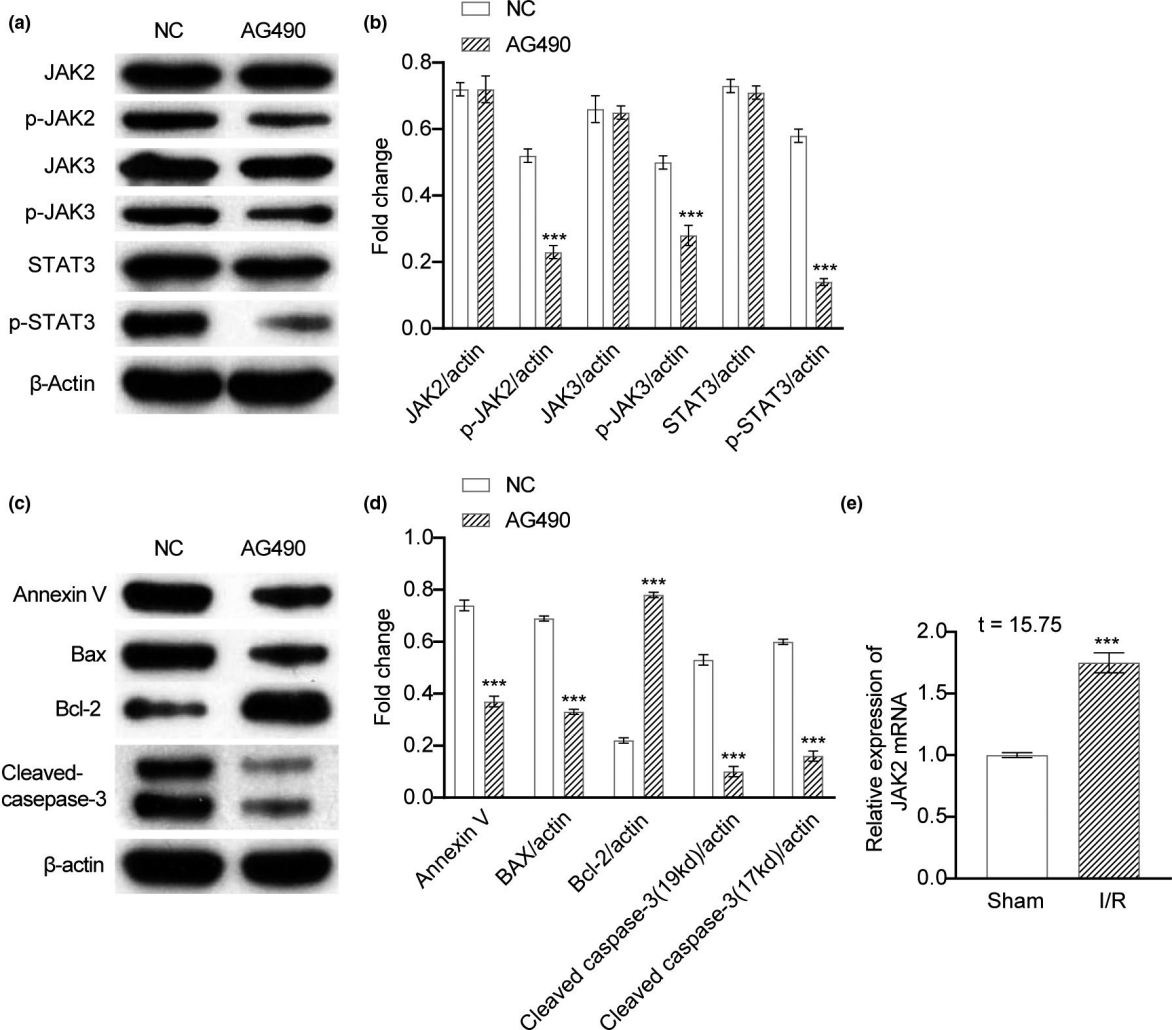
AG490 regulated the phosphorylation of JAK2/3 and STAT3. (a). AG490 regulated the phosphorylation of JAK2/3 and STAT3 determined by Western blot in the treatment of NC or AG490. (b). The fold change analysis of (a). (c). Protein expressions of apoptosis‐related proteins of Annexin V, Bax, Bcl‐2, and cleaved caspase‐3 in the treatment of NC or AG490. (d). The fold change analysis of (c). (e). mRNA expressions of JAK2 in sham mice or I/R mice. Unpaired *t* test was utilized for the comparison between the NC and the AG490. *p < .05, **p < .01, ***p < .001 compared with NC. The experiment was repeated three times independently

As is known that JAK kinases play central roles in JAK‐STAT transduction, we treated mice with AG490 in vivo to observe the changes (*p* < .05) in JAK‐STAT molecules. We hope to explore whether AG490 can regulate the phosphorylation of JAK2/3 and STAT3. As shown in Figure 2a,b, AG490 regulated the phosphorylation of JAK2/3 and STAT3 through Western blot. It is well known that JAK2/3 could mediate cell apoptosis. Therefore, we next performed Western blot to clarify the effect of AG490 on the expression of different apoptosis‐related proteins. Figure 2c,d shows that the treatment of AG490 can upregulate the expressions of antiapoptotic protein of Bcl‐2. It also can downregulate the expressions of pro‐apoptosis‐related protein of cleaved caspase‐3 and Bax (Figure 2c,d). To further decipher the mechanism of AG490 underlying apoptosis and subsequent neural damage in the MCAO model, we detected the target of AG490 JAK2 and found that JAK2 mRNA was highly expressed after MCAO (*p* < .05), respectively (Figure 2e). It was obvious that AG490 regulated the phosphorylation of JAK2/3 and STAT3.

### 3.3 AG490 exposure modulated different apoptosis‐related genes including upregulating antiapoptotic Bcl‐2 and downregulating Bax in vitro

**Figure 3:**
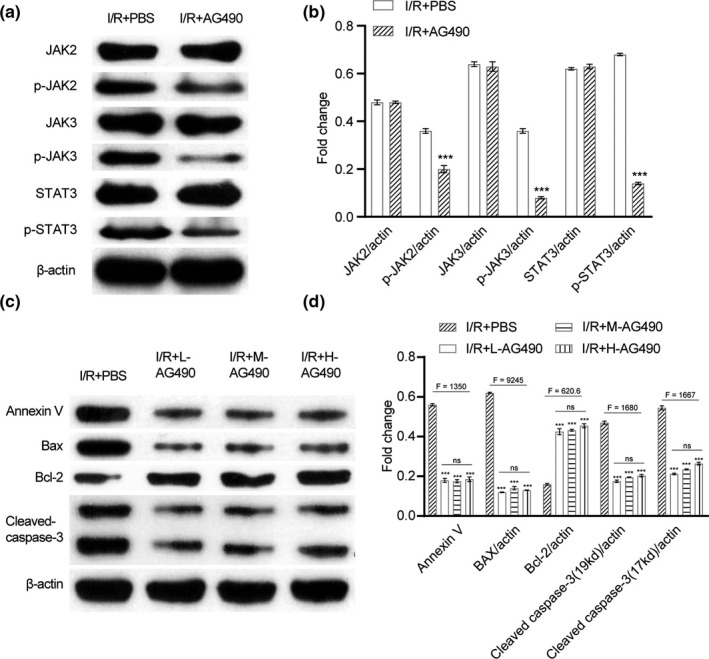
AG490 exposure modulated different apoptosis‐related genes including upregulating antiapoptotic Bcl‐2 and downregulating Bax in vitro. (a) Western blotting for protein expressions of JAK2, JAK3, p‐JAK2, p‐JAK3, STAT3, and p‐STAT3 under the treatment of I/R + PBS or I/R + AG490. (b). The fold change analysis of (a). (c). Western blotting for protein expressions of Annexin V, Bax, Bcl‐2, and cleaved caspase‐3 under the treatment of I/R + PBS, I/R + low concentration of AG490, I/R + middle concentration of AG490, and I/R + high concentration of AG490. (d). The fold change analysis of (c). Unpaired *t* test was utilized for the comparison between the I/R + PBS and the I/R + AG490. One‐way ANOVA was used among multiple groups. *n* = 5 for each mouse group. ns, not significant; *p < .05, **p < .01, ***p < .001 compared with I/R + PBS

In the neuron treated by drugs, we also observed consistent results in apoptosis induction. According to the Western blot results, AG490 exposure modulated different apoptosis‐related genes. It can upregulate antiapoptotic Bcl‐2 and downregulate Bax, cleaved caspase‐3, and Annexin V with mediation on JAK2/3‐STAT3 (Figure 3a–d). There is no dosage‐dependent effect in vitro. AG490 effectively inhibits cell apoptosis.

### 3.4 AG490 displayed antiapoptotic activity

**Figure 4:**
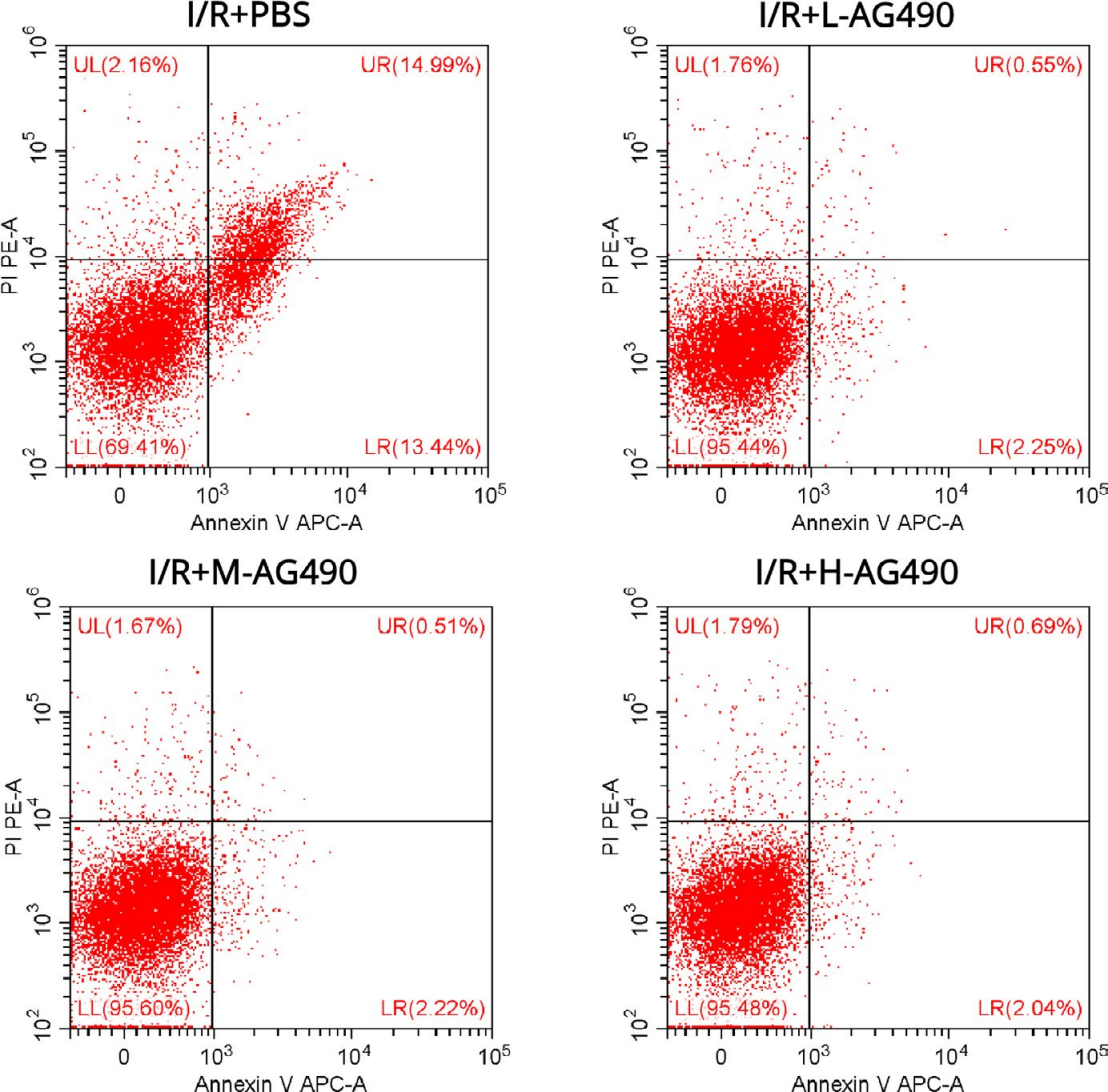
AG490 displayed antiapoptotic activity. Flow cytometry was utilized to examine the cell apoptosis under different treatments of I/R + PBS (a), low concentration of AG490 (b), middle concentration of AG490 (c), or high concentration of AG490 (d)

AG490 is a JAK/STAT inhibitor, especially applicable to JAK2‐mediated apoptosis. We examined the apoptosis rate using flow cytometry. It was proved that AG490 displayed antiapoptotic activity. Compared with the PBS group, the apoptosis of AG490 cells in the low concentration, medium concentration, or high concentration group was significantly decreased (*p* < .05) (Figure 4). And there was no significant difference between the low, medium, and high concentrations of AG490 groups (Figure 4). It also supported that AG490 exerted protection effects by preventing cell apoptosis in vitro.

### 3.5 AG490 served as a pluripotent drug for the neuroprotection in addition to its antiapoptotic activity

**Figure 5:**
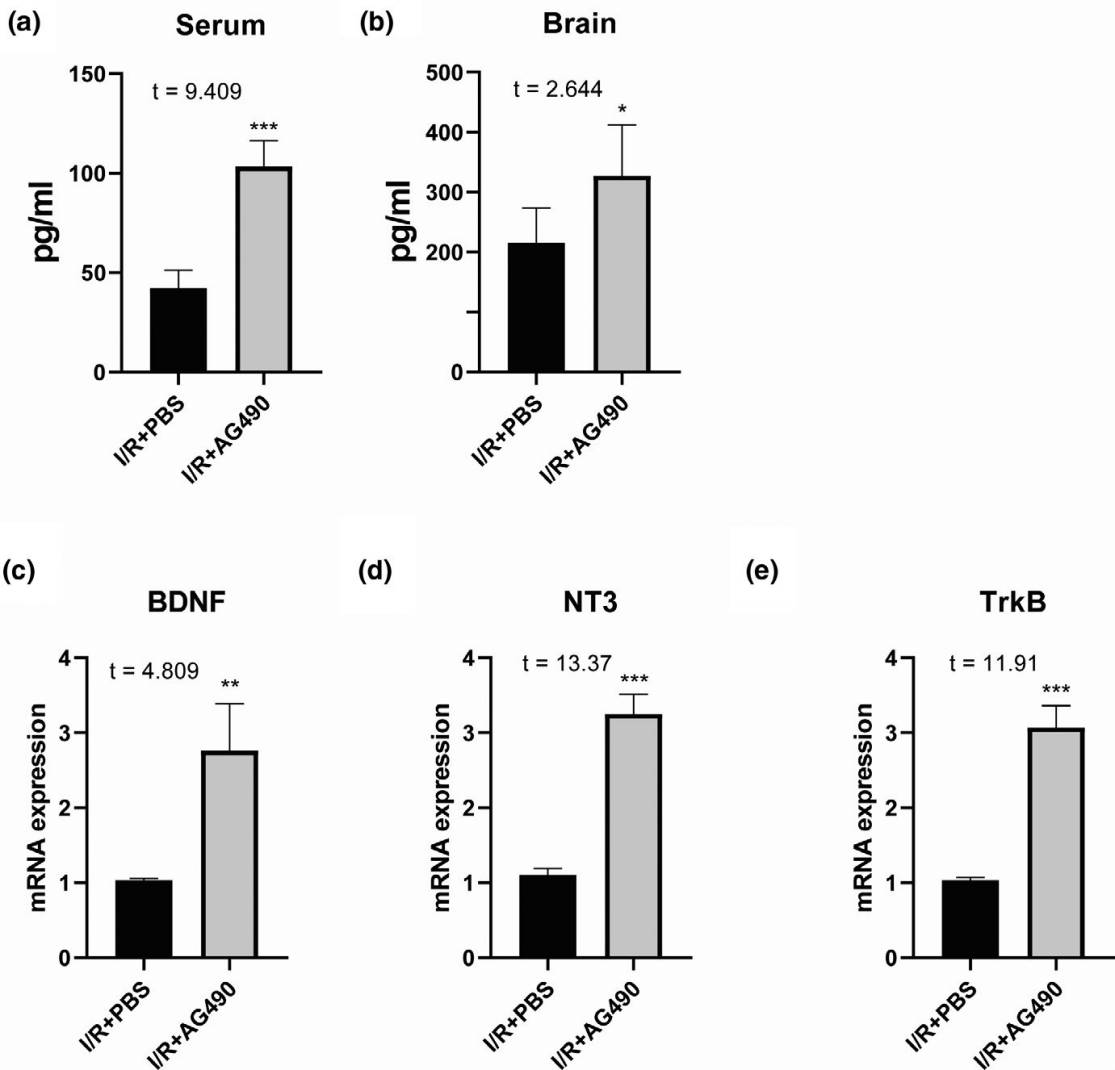
AG490 served as a pluripotent drug for the neuroprotection in addition to its antiapoptotic activity. AG490 could increase the BNDF level from serum (a) and brain tissue of MCAO mice (b). In the brain tissue, we found the higher expression of BNDF (c), neurotrophin‐3 (NT3) (d), and the neurotrophin receptor TrkB (e) in vivo. Unpaired *t* test was utilized for the comparison between the I/R + PBS and the I/R + AG490, *n* = 5 for each mouse group. *p < .05, **p < .01, ***p < .001 compared with I/R + PBS

Besides the effect of AG490 on neuroprotection against apoptosis, we also observed the alternations of neurotrophins mediated by the inhibitor for neural repairment. The results from ELISA tests revealed that the i.p. injection of AG490 could increase the BNDF level from serum and brain tissue of MCAO mice (Figure 5a,b). Meanwhile, we found the higher expression of BNDF (*p* < .05), neurotrophin‐3 (NT3), and also the neurotrophin receptor TrkB in the brain tissue (Figure 5c–e) in vivo. This evidence suggests that AG490 served as a pluripotent drug for neuroprotection in addition to its antiapoptotic activity.

## 4 DISCUSSION

AG490 is a type of tyrosine kinase receptor inhibitor that suppresses the phosphorylation and activity of STAT3 as a specific JAK inhibitor (De Vos et al., 2000; Huang et al., 2006; Nielsen et al., 1997). It was found to regulate both autoimmune diseases and carcinomas. For instance, administration of 1 mg of AG490 daily significantly decreased the severity of experimental allergic encephalomyelitis (EAE) (Zhao et al., 2017). Inhibition of STAT3 by AG490 also decreased the invasion of human pancreatic cancer cells in vitro (Huang et al., 2006). In neural cells, AG490 conferred strong protection against cell death induced by cytokines and peroxides via targeting JAK2/STAT3 or JAK3/STAT3. Indeed, JAK‐STAT bears signals from the cell membrane to the nucleus in response to extracellular growth factors and cytokines, which is important in the ischemia/reperfusion injury (Rawlings et al., 2004). This pathway is reported to be essential for protection against myocardial ischemia/reperfusion injury (Bolli et al., 2003).

Signal transduction through JAK1 and STAT3 was activated in the astrocytes during transient focal cerebral ischemia in mice, involving JAK1 kinase activity and STAT3 nuclear translocation (Booz et al., 2002; Mascareno et al., 2001). In transient focal cerebral ischemia in the mice, AG490 acted as JAK2 selective antagonist and reversed the beneficial antiapoptotic effects of sevoflurane postconditioning via JAK2 and STAT3. It suggests that the JAK‐STAT pathway may be involved in the antiapoptotic mechanism of sevoflurane. However, AG490 administration in the MCAO model is different from the reported data of using i.p. injection. Besides, the ischemia time is distinct as MCAO induced ischemia/refusion. It was clearly observed that although the phosphorylation of JAK/STAT is up/downregulated, AG490 displayed antiapoptotic activity during MCAO.

In a previous study, caspase‐1 activation‐induced pyroptosis is considered to be a vital innate immune mechanism against infection (Miao et al., 2010). Caspase‐1 is responsible for caspase‐3 activation‐mediated apoptosis and for GSDMD‐mediated pyroptosis (Shi et al., 2015). However, caspase‐3 only drives cleaved caspase‐3 for apoptosis independent of caspase‐1 in the AIM2 inflammasome response (Sagulenko et al., 2013). More evidence indicated that caspase‐3 was associated with the NLRC4 inflammasome responsible for apoptotic pathway in macrophages (Man et al., (2013)). Thus, caspase‐3 is the central caspase member for apoptosis especially in inflammation. In fact, caspase‐3 has dual role in cell death, mediating both receptor‐mediated apoptosis and necroptosis (Lee et al., 2018). Caspase‐3 also functions in maintenance and homeostasis of the adult T‐cell population (Cesaire et al., 2006). However, we found that caspase‐3 on the cerebral tissue was inhibited in our current data, which are using series of specific inhibitors in the JAK‐STAT pathways. It indicated that AG490 confers protection against neural apoptosis during the process of cerebral ischemia/reperfusion injury. This was consistent with the in vitro data of AG490 on cultured neurons, upon apoptosis induction. Furthermore, we speculated that AG490 mainly targets the injured neurons instead of the infiltrated inflammatory cells in vivo. This infiltration was reduced, whereas the antiapoptotic effect by caspase‐3 inhibition might protect these cells.

In other parameter detection in vivo, we found that expression of neurotrophins was affected by post‐treatment of AG490 with i.p. injection. Brain‐derived neurotrophic factor (BDNF) plays a pivotal role in neural development and diseases (Binder & Scharfman, 2004). It was reported that BDNF mRNA expression was higher in the I/R model in vivo from 6 hr to 3 days post‐I/R surgery (Li et al., 2018). Here, we examined that AG490 treatment further enhanced the expression of BDNF not only in the serum but also in the brain. This protein level was consistent with its transcriptional level. In addition, NT3 is a neurotrophic factor related to NGF and BDNF. Primary mouse fibroblasts genetically modified to produce NT‐3 were grafted to promote corticospinal axonal growth and partial functional recovery after spinal cord injury, suggesting the neural protective function of NT‐3.

In our current data, AG490 i.p. injection but not cerebral delivery also triggered the mRNA expression of NT‐3 in the brain, suggesting that AG490 could penetrate to CNS for transcriptional regulation of series of neurotrophins in vivo. It was reported that animals presented a massive opening of blood–brain barrier after MCAO indicated by sucrose uptake method, and the permeability of the BBB could be reduced by MMP inhibitor (Cen et al., 2013). Here, we found that AG490 i.p. administration after I/R operation was responsible for the gene expression of cerebral NT‐3. Another key neurotrophic factor modulated by AG490 in the CNS is the TrkB, which was identified as receptor for BDNF and NT‐3. TrkB is also a tyrosine kinase with confined expression on central and peripheral nervous systems (Yamada & Nabeshima, 2003). Targeted disruption of the TrkB neurotrophin receptor gene results in nervous system lesions and neonatal death (Klein et al., 1993). However, the transcription of TrkB in the CNS was enhanced after AG490 intervention. Our data revealed that AG490‐mediated TrkB could serve as a signaling receptor for the increased neurotrophins.

As a limitation of this study, we did not set up the sham surgery group, which could work as a critical control to eluate the possible side effects of AG490 in the brain. In the future work, we will perform further in vivo experiments with the sham surgery group.

## 5 CONCLUSION

Therefore, AG490 is pluripotent for cerebral ischemia/reperfusion injury through both antiapoptosis and neuroprotective activities, and the former effect is dependent on its regulation of the JAK‐STAT pathway.

## CONFLICT OF INTEREST

The authors declare that they have no conflict of interest.

## AUTHOR CONTRIBUTIONS

Lichao Fan and Lichun Zhou designed the study, performed the research, analyzed data, and wrote the paper.

### Peer Review

The peer review history for this article is available at https://publons.com/publon/10.1002/brb3.1911.

## Data Availability

The authors confirm that all data underlying the findings are fully available without restriction. All relevant data are within the paper.
